# Cell-selective oligonucleotide STAT3 inhibitor for immunotherapy of human acute myeloid leukemia

**DOI:** 10.1186/2051-1426-3-S2-P362

**Published:** 2015-11-04

**Authors:** Qifang Zhang, Sakib Hossain, Dayson Moreira, Priyanka Duttagupta, Xingli Zhao, Piotr Swiderski, Marcin Kortylewski

**Affiliations:** 1 City of Hope, Duarte, CA, USA; 2 Beckman Res. Inst. City of Hope, Duarte, CA, USA

## 

STAT3 transcription factor is persistently activated in cancer cells and in diverse tumor-associated immune cells being an important oncogene and an essential immune checkpoint regulator. It is a highly desirable but challenging therapeutic target for pharmacological intervention. We previously demonstrated that ligand for the intracellular receptor TLR9 (CpG ODN) allows for the uptake and cytoplasmic delivery of siRNA into specific target cells[1-3]. Now, we used this approach to deliver decoy oligodeoxynucleotides (dODNs) to prevent STAT3 DNA binding and transcriptional activation in variety of mouse and human target cells. These include immune cells (dendritic cells, B cells and MDSCs) and certain types of cancer cells, such as acute myeloid leukemia (AML) and B cell lymphoma. After uptake, CpG-STAT3dODN molecules bind and sequester activated STAT3 proteins, thereby inhibiting their transcriptional activity. CpG-STAT3dODN induced apoptosis in STAT3-dependent cancer cells without affecting the viability of immune cells. In addition, the concomitant STAT3-blocking/TLR9-triggering augmented immunogenicity of patients' derived AML cells. CpG-STAT3dODN treatment reduced expression of immunosuppressive enzyme, arginase-1, in leukemic cells while upregulating levels of HLA-DR and costimulatory molecules and partly restoring proliferation of co-cultured autologous T cells. The chemically modified CpG-STAT3dODNs have improved resistance to serum degradation. As shown by our studies using disseminated human MV4-11 AML, repeated i.v. injections of CpG-STAT3dODN reduced levels of activated STAT3 in target cells with ED_50_ ~2.5 mg/kg and within two weeks resulted in AML regression. To assess the combined antitumor effect of STAT3-blocking/TLR9-triggering in immunocompetent host, we used syngeneic *Cbfb/MYH11* AML. The i.v. administration of CpG-STAT3dODN over two weeks resulted in long-term survival of the majority of mice. These potent antitumor effects resulted from the increased immunogenicity of leukemic cells rather than host antigen-presenting cells as suggested by the comparable extent of tumor regression in wild-type and *TLR9*-deficient mice. These finding support further development of CpG-STAT3dODN strategy for treatment of AML and potentially B cell lymphoma.

This project described was supported by the National Cancer Institute of the National Institutes of Health under award number R01CA155367 to M.K.
